# A review on regulation of DNA methylation during post-myocardial infarction

**DOI:** 10.3389/fphar.2024.1267585

**Published:** 2024-02-13

**Authors:** Wenqiang Han, Wenxin Wang, Qinhong Wang, Kellina Maduray, Li Hao, Jingquan Zhong

**Affiliations:** ^1^ National Key Laboratory for Innovation and Transformation of Luobing Theory, The Key Laboratory of Cardiovascular Remodeling and Function Research, Chinese Ministry of Education, Chinese National Health Commission and Chinese Academy of Medical Sciences, Department of Cardiology, Qilu Hospital of Shandong University, Jinan, China; ^2^ Department of Gerontology, The First Affiliated Hospital of Shandong First Medical University and Shandong Provincial Qianfoshan Hospital, Jinan, China; ^3^ Department of Cardiology, Qilu Hospital (Qingdao), Cheeloo College of Medicine, Shandong University, Qingdao, China

**Keywords:** myocardial infarction, DNA methylation, inflammation, autophagy, proliferation, fibrosis

## Abstract

Myocardial infarction (MI) imposes a huge medical and economic burden on society, and cardiac repair after MI involves a complex series of processes. Understanding the key mechanisms (such as apoptosis, autophagy, inflammation, and fibrosis) will facilitate further drug development and patient treatment. Presently, a substantial body of evidence suggests that the regulation of epigenetic processes contributes to cardiac repair following MI, with DNA methylation being among the notable epigenetic factors involved. This article will review the research on the mechanism of DNA methylation regulation after MI to provide some insights for future research and development of related drugs.

## 1 Introduction

Cardiovascular disease (CVD) is the leading cause of death worldwide and poses a significant medical and economic burden on countries worldwide (Han, 2019; Sturgeon et al., 2019; Zhao et al., 2019; Townsend et al., 2022). Among CVD events, myocardial infarction (MI) has been shown to contribute significantly to disease burden and mortality (Mathers and Loncar, 2006; White and Chew, 2008; Heidenreich et al., 2013). Following MI, cardiac repair involves a complex series of events, including an inflammatory phase, repair and proliferation phase, and mature phase (Prabhu and Frangogiannis, 2016). The inflammatory phase, initiated by tissue damage such as cell necrosis, triggers the recruitment of immune cells that generate a robust inflammatory response to remove damaged cells and extracellular matrix components (Frangogiannis, 2012). Transitioning from the inflammatory phase to the repair and proliferation phase requires the activation of multiple inhibition pathways to reduce post-infarction inflammation, involving a variety of cell types and extracellular matrix components such as neutrophils, monocyte-macrophage systems, and endothelial cells. The success of this transition is critical for effective repair of the infarcted area (Frangogiannis, 2012; Kain et al., 2014). During the repair and proliferation phase, inflammation gradually subsides, and fibroblasts transform into myofibroblast phenotypes, leading to a series of fibrotic reactions that culminate in scar formation and neovascularization in the mature phase (Nahrendorf et al., 2010). Despite significant progress in understanding the pathogenesis of MI and the development of novel treatments such as reperfusion therapy and drug therapy, treating MI patients remains challenging (Saleh and Ambrose, 2018). Identifying key molecular targets, such as those involved in apoptosis, autophagy, inflammation, and fibrosis, that occur successively after MI may lead to the development of more effective treatment strategies in the future.

**FIGURE 1:**
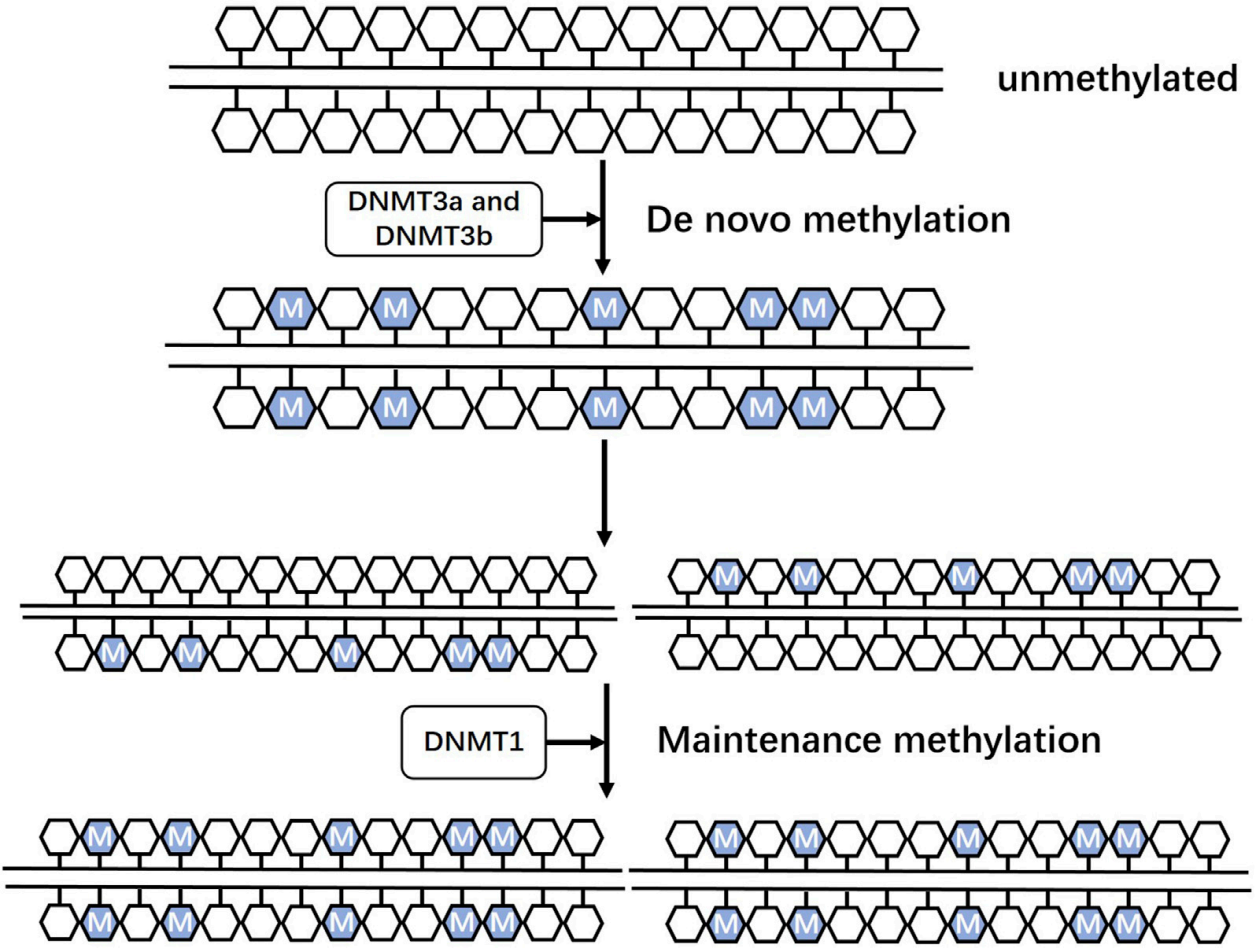
Roles of three DNA methyltransferases Dnmt3a and DNMT3b are responsible for *de novo* methylation during development by establishing a novel methylation pattern on unmodified DNA. DNMT1 conveys DNA methylation patterns during DNA replication of the newly synthesized daughter strand of the parental strand and plays a role in maintaining post-replicative methylation in cells.

DNA methylation is a well-known epigenetic modification in mammalian genomes that is regulated by a family of DNA methyltransferases (DNMTs). DNMTs catalyze the transfer of a methyl group from s-adenine methionine to a cytosine residue at position 5C to form 5 mC (Moore et al., 2013). Dnmt3a and DNMT3b are responsible for *de novo* methylation (Figure 1) during development by establishing a novel methylation pattern on unmodified DNA (Okano et al., 1999). DNMT1 conveys DNA methylation patterns during DNA replication of the newly synthesized daughter strand of the parental strand and plays a role in maintaining post-replicative methylation in cells (Li et al., 1992; Sen et al., 2010; Moore et al., 2013). However, some studies argue for a cooperative role of the three DNMTs during DNA methylation (Fatemi et al., 2002; Kim et al., 2002). DNA methylation predominantly occurs at palindromic cytosine phosphate guanine dinucleotides (CpGs) in the human genome, with 60%–80% of approximately 28 million CpGs usually being methylated (Smith and Meissner, 2013). CpG islands (CpGis), known as CG dense regions, account for less than 10% of CpGs and are located in promoter regions of approximately 70% of annotated genes, including nearly all housekeeping genes, tissue-specific genes, and developmentally regulated genes, to regulate transcription (Larsen et al., 1992; Saxonov et al., 2006; Zhu et al., 2008; Deaton and Bird, 2011). DNA methylation can directly interfere with the binding of specific transcription factors or indirectly bind specific transcriptional repressors, such as methyl CpG binding protein (MeCP) and methyl CpG binding domain protein, to repress chromatin transcriptional states and mediate gene silencing (Iguchi-Ariga and Schaffner, 1989; Comb and Goodman, 1990; Falzon and Kuff, 1991; Prendergast et al., 1991; Hendrich and Tweedie, 2003; Das and Singal, 2004; Russell-Hallinan et al., 2018). However, DNA methylation may play different roles depending on the genomic region sequences (Moore et al., 2013). Intergenic regions, for instance, serve to suppress potentially harmful genetic factors (Walsh et al., 1998; Gaudet et al., 2004), while DNA hypermethylation within gene bodies is linked to increased gene expression (Hellman and Chess, 2007; Ball et al., 2009; Aran et al., 2011).

While DNA methylation was initially considered a long-lasting and stable epigenetic modification, current research indicates that it is a dynamic and reversible biological process (Fardi et al., 2018; Russell-Hallinan et al., 2018). The process may be mediated by ten-eleven translocation (Tet) enzymes, namely Tet1, Tet2, and Tet3. Tet1, in particular, introduces a hydroxyl group to the methyl group at the 5 mC site, resulting in the formation of 5 hmC (Tahiliani et al., 2009; Ito et al., 2010). Once formed, 5 hmC may be converted to 5 fC and 5 caC under Tet enzyme catalyzed oxidation, followed by decarboxylation to cytosine (Ito et al., 2011). 5 fC and 5 caC can also be excised by thymine DNA glycosylase and subsequently subjected to pathways such as base excision repair to remove methylation modifications from cytosine (Kohli and Zhang, 2013). There is also supporting evidence indicating that akin to methylation, 5 hmC can influence gene expression, leading to notable decreases in the binding affinity of MeCP2 (Valinluck et al., 2004).

Research has demonstrated alterations in the degree of methylation of certain genes in individuals with myocardial infarction (Ek et al., 2016; Rask-Andersen et al., 2016; Ward-Caviness et al., 2018; Thunders et al., 2019), which are associated with cardiac function, cardiogenesis, and recovery after ischemic injury. To understand the early dynamic changes of gene expression after MI, RNA-seq and MeDIP-seq were used on heart tissues from AMI mice at different time points (Han et al., 2022; Luo et al., 2022), and the results showed that DNA methylation plays an important role in the pathophysiological progression after MI. We reviewed the existing research and elucidated the role of DNA methylation regulation in inflammation, fibrosis and other aspects after myocardial infarction.

## 2 Roles of DNA methylation after myocardial infarction

### 2.1 Inflammation and DNA methylation

After myocardial infarction, hypoxia often leads to endothelial cell barrier impairment and increased vascular permeability due to decreased adenylate cyclase activity and intracellular cAMP levels. This promotes leukocyte infiltration and activates the death process of myocardial cells, primarily cell necrosis, accompanied by apoptosis and autophagy (Ogawa et al., 1992; Hotchkiss et al., 2009; Nahrendorf et al., 2010; Eltzschig and Eckle, 2011; Kain et al., 2014; Prabhu and Frangogiannis, 2016). Immediate reperfusion treatment releases soluble inflammatory mediators that recruit neutrophils, which directly damage endothelial cells by producing reactive oxygen species (ROS), cytokines, proteases, and lipids. These further increases leukocyte adhesion and damages the tight junctions between cells, exacerbating endothelial cell barrier dysfunction (Ma et al., 1993; Weyrich et al., 1993; Vinten-Johansen, 2004; Arslan et al., 2011; Timmers et al., 2012). Endogenous molecules released by damaged or dead cells and extracellular matrix, called danger-associated molecular patterns (DAMPs), initiate immune responses after MI, such as high-mobility group box-1 (HMGB1), heat shock proteins (HSPs), and fibronectin fragments (Fan et al., 2005; Timmers et al., 2012; Mi et al., 2019; Gong et al., 2020). These molecules continuously activate pattern recognition receptors (PRRs), such as Toll-like receptors (TLRs), in monocytes and neutrophils of innate immunity. The activation of these molecules initiates intracellular signal transduction pathways, such as mitogen-activated protein kinase (MAPK), Janus kinase (JAK), and calcineurin pathway. This induces the cascade amplification of nuclear factor-kappaB (NF-κB) and activating protein AP-1 to downstream inflammatory mediators (Mann, 2011; Newton and Dixit, 2012; Ghigo et al., 2014). An excessive inflammatory response may lead to pathological phenomena, such as maladaptive remodeling, interstitial fibrosis, and impaired myocardial contractility. Therefore, the recruitment of inflammatory-related cells must be strictly controlled to ensure functional cardiac healing. For instance, secreting growth differentiation factor-15, transforming growth factor-β (TGF-β), and other anti-inflammatory signals can limit the infiltration range of immune cells (Wilson et al., 2004; Wrigley et al., 2011; Ghigo et al., 2014).

**FIGURE 2:**
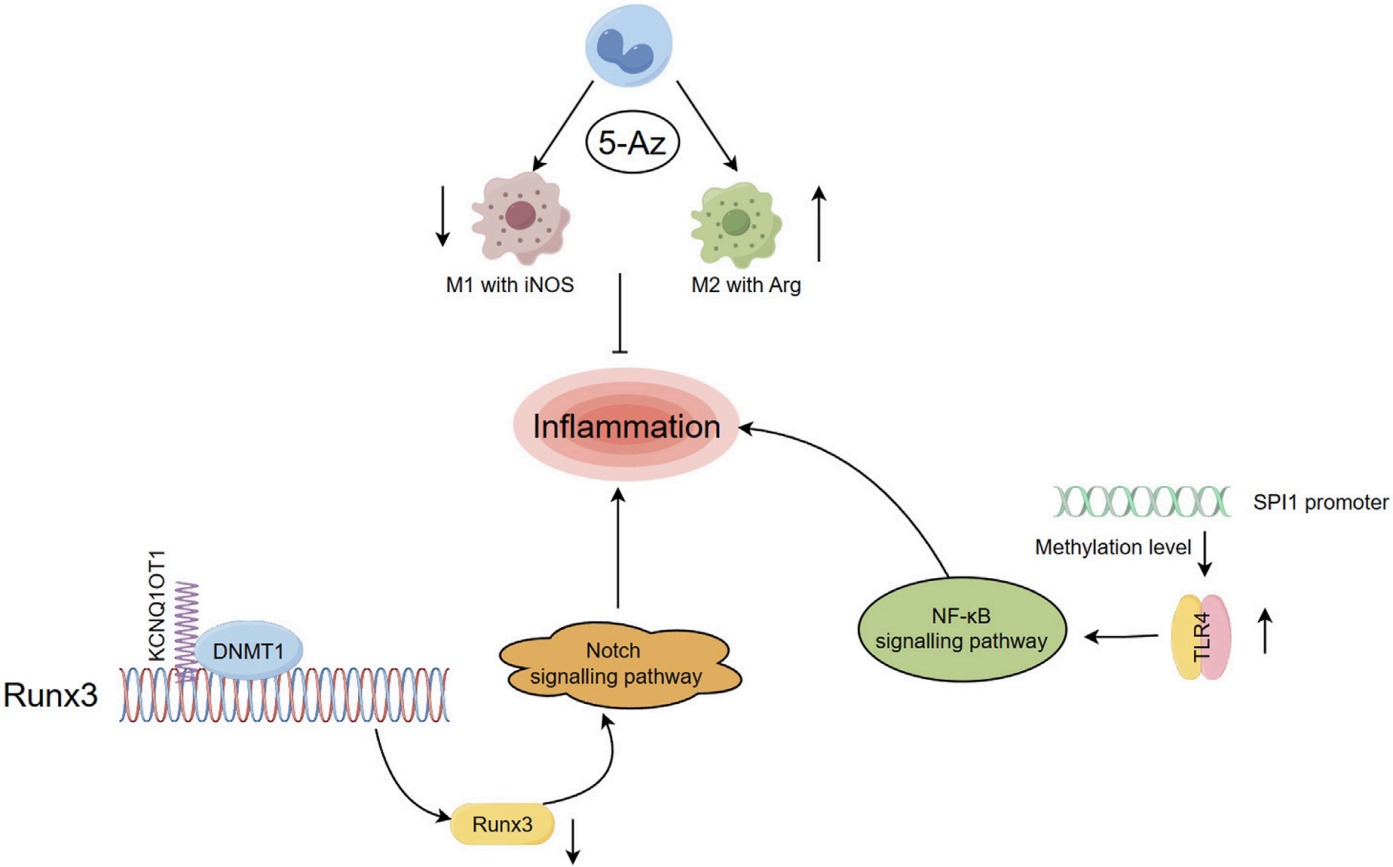
Regulation of DNA methylation on inflammation after myocardial infarction (Han, 2019) Rats with MI were administered with 5-Az, resulting in a decrease in the number of M1 phenotype macrophages expressing iNOS and an increase in the level of anti-inflammatory M2 phenotype macrophages. (Zhao et al., 2019). lncRNA KCNQ1OT1 mediated imprinted gene silencing by recruiting DNMT1 and elevating the CpGi methylation level in the promoter region of RUNX3, which in turn suppressed RUNX3 expression levels and affected inflammatory responses through the Notch pathway (Townsend et al., 2022). After MI, the levels of DNA methylation at the SPI1 promoter CpGi were significantly decreased, upregulated SPI1 expression levels bound to the TLR4 promoter and activated the TLR4/NF-κB pathway, exacerbating inflammation.

The initial response of immune cells to damage is regulated by immediate epigenetic modifications, such as DNA methylation-mediated key activation. However, limited research has been conducted on the epigenetic modification of immune cells during the later stages, particularly during the repair regression phase (Busslinger and Tarakhovsky, 2014; Phan et al., 2017; Placek et al., 2019). In an experiment, rats with MI were administered with 5-azacytidine (5-AZ) as a DNA methyltransferase inhibitor (DNMTi). This intervention led to a reduction in the count of M1 phenotype macrophages expressing iNOS and an elevation in the presence of anti-inflammatory M2 phenotype macrophages in the infarcted myocardium (Figure 2). The expression frequency of Arg1 increased, improving the myocardial diastolic function after MI (Stresemann and Lyko, 2008; Kim et al., 2014). Dynamic DNA methylation modification plays a significant role in the self-renewal and differentiation of immune cells, presenting itself as a potential target for addressing inflammatory processes in diseases (Luo et al., 2018; Morales-Nebreda et al., 2019).

Studies have demonstrated that, in addition to coding genes, long noncoding RNAs (lncRNAs) play a role in the progression of genetic variations following MI. They are involved in regulating cardiac development and pathological conditions, such as myocardial ischemia (Saddic et al., 2017). For example, the lncRNA potassium voltage-gated channel subfamily Q member 1 overlapping transcript 1 (KCNQ1OT1) is expressed at significantly higher levels in MI patients compared to healthy individuals (Vausort et al., 2014). KCNQ1OT1 is responsible for silencing a gene cluster in cis and is associated with epigenetic modifications such as DNA methylation and histone modifications that play a role in colorectal carcinogenesis (Nakano et al., 2006; Wan et al., 2013). Previous research has shown that knockdown of KCNQ1OT1 can modulate adiponectin receptor 1 and attenuate ischemia-reperfusion (I/R) injury after acute myocardial infarction (AMI) by affecting the p38 MAPK/NF-κB signaling pathways (Li et al., 2017). In a study on MI mice, it was found that lncRNA KCNQ1OT1 mediated imprinted gene silencing by recruiting DNMT1 and elevating the CpGi methylation level in the promoter region of runt related transcription factor 3 (RUNX3), which in turn suppressed RUNX3 expression levels. This affected cardiac microvascular endothelial cells activity and inflammatory responses in post-MI mice through the Notch1 pathway (Figure 2), and the dysfunction of endothelial cells was associated with adverse cardiac remodeling after MI (Mohammad et al., 2010; Wang Y. et al., 2019). Furthermore, when RUNX3 is knocked down as a common downstream target of TGF-β and Notch signal pathways in cardiovascular disease, it can attenuate hypoxia-induced endothelial-mesenchymal transition (EndMt) and reverse human cardiac microvascular endothelial cells function. This suggests that targeting RUNX3 may be a novel approach to mediating EndMt for the treatment of CVD (Liu et al., 2017).

The proto-oncogene SPI1 is considered a key player in the progression to heart failure (HF) after MI. It is tightly associated with inflammation, immune activity, and apoptosis, and its possible target TLR4 plays an equally important role in mediating tissue damage and inflammatory responses (Niu et al., 2019; Ramirez-Carracedo et al., 2020). A recent study showed that in MI mice and hypoxia-exposed HL-1 cells, the levels of DNA methylation at the SPI1 promoter CpGi were significantly decreased, leading to the activation of SPI1 transcription. Upregulated SPI1 expression levels then bound to the TLR4 promoter and activated the TLR4/NF-κB pathway, exacerbating inflammation and cardiomyocyte apoptosis (Liu and Huang, 2022) (Figure 2). Previous studies have confirmed that the activation of the TLR4/NF-κB signaling pathway is involved in inducing tissue injury and inflammation after MI. Inhibition of this pathway is a promising and controllable target for anti-inflammatory treatment after MI (Li et al., 2018; Wang XZ. et al., 2019; Liu et al., 2019; Li et al., 2020).

During different stages after MI, immune cells exhibit functional heterogeneity. In the early stages of myocardial injury, functional changes involving macrophages are accompanied by infiltration of neutrophils and monocytes that promote inflammation. Later on, repair-predominant subpopulations gradually replace these cells (Bajpai et al., 2019; Dick et al., 2019). Further exploration of cell-specific functional type changes during inflammation, linked to spatiotemporally specific epigenetic modifications such as DNA methylation, is necessary to identify the best targets for clinical treatment after MI.

### 2.2 Autophagy and DNA methylation

As a dynamic circulatory system, autophagy primarily transports long-lived proteins and damaged organelles into lysosomes for degradation (Yang and Klionsky, 2010; Mizushima and Komatsu, 2011). Autophagy is often associated with ischemic heart disease (Bravo-San Pedro et al., 2017) and can be further enhanced by I/R (Valentim et al., 2006). During acute ischemia, autophagy may play a protective role in maintaining energy supply and reducing infarct size (Matsui et al., 2007; Zhu et al., 2007; Kanamori et al., 2011; Chen et al., 2013), while also endowing the body with some anti-stress ability (Yan et al., 2006). However, excessive activation of autophagy by oxidative stress during reperfusion (Liu et al., 2018), which clears damaged organelles intracellularly, can be harmful to the organism. Autophagy is a controversial topic, as many studies have shown that autophagy, as a stress response mechanism, is upregulated in myocardial reperfusion injury, and the impairment of autophagy clearance ability can lead to cell death (Ma et al., 2012). Therefore, balancing autophagy activity in a timely manner to keep it within a safe range is crucial to maintain the stability of myocardial cells and overall cardiac structure and function (Nakai et al., 2007; Xu et al., 2015) and prevent cardiac dysfunction after MI. Currently, there is no effective intervention strategy for regulating autophagy-related cardiovascular diseases in clinical practice (Zhou et al., 2019). However, epigenetic modifications such as DNA methylation have shown some therapeutic prospects in regulating autophagy after MI.

**FIGURE 3:**
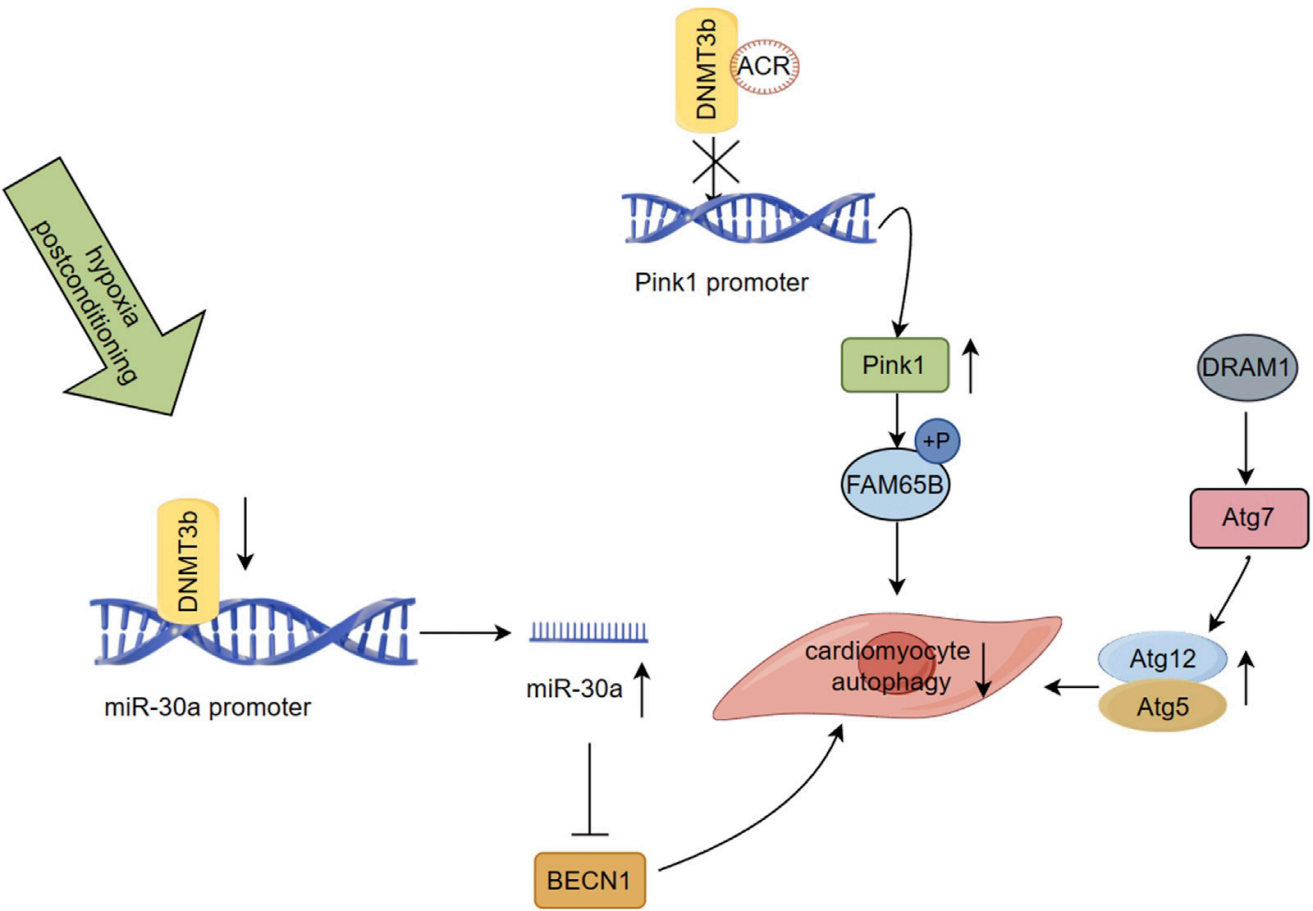
Regulation of DNA methylation on autophagy after myocardial infarction (Han, 2019) ACR binds to DNMT3b and prevents DNA methylation of the Pink1 promoter, which activates the expression of the Pink1, and it mediates FAM65B phosphorylation to inhibit autophagy and cell death (Zhao et al., 2019). Suppressed DNMT3b activity leads to miR-30a hypomethylation and increases its expression, which results in the targeting of the autophagy related gene BECN1 by miR-30a, inhibiting autophagy induction in aging cardiomyocytes (Townsend et al., 2022). DRAM1 was found to be negatively regulated in the rat model of AMI. Regulating the autophagic flow of DRAM1-Atg7-Atg12/Atg5 under the stress condition of myocardial ischemia can alleviate autophagic flow and regulating the mechanism of myocardial cell protection.

Poorly regulated autophagy is associated with cardiovascular diseases, including myocardial infarction (Tanaka et al., 2000; Yamamoto et al., 2000; Maejima et al., 2013). PTEN-induced putative kinase 1 (Pink1) is downregulated in advanced stages of human HF and is believed to be crucial for maintaining normal cardiac function (Billia et al., 2011). Family with sequence similarity 65 member B (FAM65B) is involved in several cellular processes, such as cell differentiation, but its role in regulating autophagy in cardiomyocytes was previously unknown (Balasubramanian et al., 2014; Diaz-Horta et al., 2014). Recently, a study revealed that autophagy-related circular RNA (ACR) directly binds to DNMT3b and prevents DNA methylation of the Pink1 promoter, a process regulated by DNMT3b. ACR activates the expression of the Pink1 promoter by targeting a reduction in its DNA methylation level. Additionally, it facilitates the phosphorylation of FAM65B, leading to the inhibition of autophagy and cell death. This process reduces ischemia/reperfusion injury and the area of myocardial infarction (Figure 3). The existing evidence demonstrates that the ACR-Pink1-FAM65B axis, which is regulated by the DNA methylation pathway, can be utilized as a regulator of cardiac autophagy (Zhou et al., 2019). Circular RNA interacts with DNMT and participates in the regulation of DNA methylation, which highlights its potential as a therapeutic target for MI and I/R injury.

Ischemic postconditioning (IPostC) is a potential strategy for protecting cardiomyocytes during early reperfusion by intermittently interrupting coronary blood flow, thereby reducing I/R injury and final infarct size (Zhao et al., 2003; Heusch et al., 2011). IPostC has been implicated in regulating autophagy and may offer myocardial protection in I/R injury (Ma et al., 2011; Wang et al., 2016). The methylation status of miRNA promoters may serve as potential epigenetic biomarkers for clinical applications and directly regulate their expression levels (Vera et al., 2017; Ortiz et al., 2018). A study investigating hypoxic postconditioning (HPostC) in aging cardiomyocytes exposed to hypoxia reoxygenation (H/R) injury revealed that DNMT3b mediates hypomethylation of the miR-30a promoter, leading to an increase in the expression levels of miR-30a. This results in the targeting of the autophagy related gene BECN1 by miR-30a, inhibiting autophagy induction in aging cardiomyocytes, and promoting HPostC to exert cardioprotective effects (Figure 3). Suppressed DNMT3b activity leads to miR-30a hypomethylation and increases its expression, providing an additional epigenetic regulatory pathway (Wang et al., 2020). This study not only uncovers the protective effect of IPostC against I/R injury in aging cardiomyocytes but also sheds light on the underlying molecular mechanism of I/R injury via autophagy regulation. It provides a highly promising DNA methylation-related therapeutic target for achieving precise treatment and improving myocardial injury in elderly patients with MI, in agreement with previous clinical reports (Wang et al., 2016; Zhang et al., 2018).

Damage-regulated autophagy modulator 1 (DRAM1) is a lysosomal membrane protein that is be beneficial in attenuating autophagic flux impairment and cardiac functional maladaptation in acute myocardial infarction (AMI) due to ischemic stress, ultimately improving cardiac outcomes in patients (Crighton et al., 2006; Wu et al., 2014; Wu et al., 2018). A recent study using an AMI rat model demonstrated that DRAM1 upregulates the mRNA level of Atg12 in cardiomyocytes *in vitro* and *in vivo*, while decreasing the levels of free Atg12 protein. Additionally, an increase in the formation of Atg12-Atg5 conjugates was identified. DRAM1 showed a significant direct interaction only with Atg7, which regulated the autophagic flow of DRAM1-Atg7-Atg12/Atg5 under the stress condition of myocardial ischemia, thereby alleviating autophagic flow and regulating the mechanism of myocardial cell protection (Wu et al., 2018) (Figure 3). Moreover, DRAM1 was found to be negatively regulated in the rat model of AMI, where ischemia-induced aberrant DNA hypermethylation status at multiple promoter CpG sites of DRAM1 was associated with decreased DRAM1 expression levels in the infarct border zone. In summary, these findings suggest that targeting DNA methylation and autophagy pathways via DRAM1 could be a promising therapeutic strategy to regulate autophagy flux and improve outcomes in patients with AMI.

In recent years, epigenetic regulation such as DNA methylation and histone modification has been extensively investigated in the context of autophagy. These modifications can not only directly modify autophagy-related genes but also influence signaling molecules that regulate these genes (Klionsky et al., 2021). In cancer research, the methylation status of autophagy-regulating effector molecules has been found to play a significant role in exhibiting biphasic regulatory effects (Liu H. et al., 2013; Liao et al., 2014; Nihira et al., 2014; Zhang et al., 2016; Bhol et al., 2020). Similarly, DNA methylation in conjunction with autophagy in the context of MI and I/R injury presents a promising avenue for research. Targeted therapy strategies based on the aberrant DNA methylation status of autophagy-related molecules in cardiomyocytes may become a crucial clinical direction in the near future.

### 2.3 Fibrosis and DNA methylation

In tissues affected by myocardial infarction, ongoing ischemia, hypoxia, inflammation, and other stimuli induce cardiac fibrosis and abnormal cardiac remodeling, which are inevitable pathological changes in MI progression (van den Borne et al., 2010; Barallobre-Barreiro et al., 2012; Opie et al., 2006; Brown et al., 2005). Interstitial fibroblasts respond to elevated TGF-β and changes in the extracellular matrix by transforming into myofibroblasts, leading to further increases in proliferative activity (Willems et al., 1994; Frangogiannis et al., 2000; Souders et al., 2009; Turner and Porter, 2013). These pathways serve as markers for the proliferative phase of repair after MI and are highly involved in regulating cardiac fibrosis-related remodeling, which has a major impact on cardiac function (Hinz et al., 2007). Under stress conditions, cardiac fibroblasts express α-smooth muscle actin (α-SMA) and the smooth muscle dedifferentiation marker myosin heavy chain embryonic isoform (Frangogiannis et al., 2000). This disrupts the equilibrium between matrix metalloproteinases (MMPs) and tissue inhibitors of metalloproteinases, leading to the production of an extracellular matrix (ECM) that distinguishes fibroblasts (Berk et al., 2007). The reparative proliferation process of the myocardium in the infarcted area involves the regulated deposition and breakdown of ECM, particularly collagen, which is controlled by regulatory elements such as MMPs (Watson et al., 2014; Olsen et al., 2017). Although fibrosis initially reflects adaptive protective mechanisms, dysregulation of the balance can lead to excessive ECM deposition, resulting in pathological reactive fibrosis that increases myocardial stiffness, disrupts tissue architecture, and impairs cardiac function (Levick et al., 2009; Qi et al., 2014; Murtha et al., 2017). Accumulating evidence suggests that the hyperactive fibrotic phenotype may be attributed to the accumulation of gene expression within the interstitium, and the causal role that epigenetics plays in fibrosis is gradually being recognized. DNA methylation may play an important role in the progression of cardiac fibrosis (Gabrielsen et al., 2007; Mann and Mann, 2013; Watson et al., 2014; Yang and Schwartz, 2015; Kale et al., 2021).

NEIL3 is a mammalian oxidized base-specific DNA glycosylase in the base excision repair pathway. It is elevated in the myocardium of patients with HF, and its expression increases significantly in fibroblast enriched fractions after myocardial infarction in mice (Liu M. et al., 2013; Olsen et al., 2017). NEIL3 regulates cell proliferation in various human tumor tissues, murine hematopoietic tissues, and various stem cell populations in mice (Torisu et al., 2005; Hildrestrand et al., 2009; Regnell et al., 2012; Reis and Hermanson, 2012; Rolseth et al., 2013). Some studies suggest that NEIL3 affects the balance between methylation and oxidative demethylation of cytosine related epigenetic modifications in the heart. These modifications include differential regulation of both 5 mC and 5 hmc. Alternatively, NEIL3 may regulate the proliferation and differentiation of fibroblast-like cells during post-MI repair by affecting DNA methylation sites. The results show that NEIL3 deficient mice have a decreased ability to regulate cell proliferation after MI, implying a deregulation of fibroblast-like cells proliferation and differentiation. This leads to a disruption of the extracellular matrix balance, including increased MMP2 production and consequent fatal consequences such as myocardial rupture. Therefore, understanding the regulatory mechanisms of fibroblast-like cell proliferation and differentiation and ECM balance is important for developing clinical antifibrotic drugs. NEIL3 could be a potential therapeutic target for this purpose.

**FIGURE 4:**
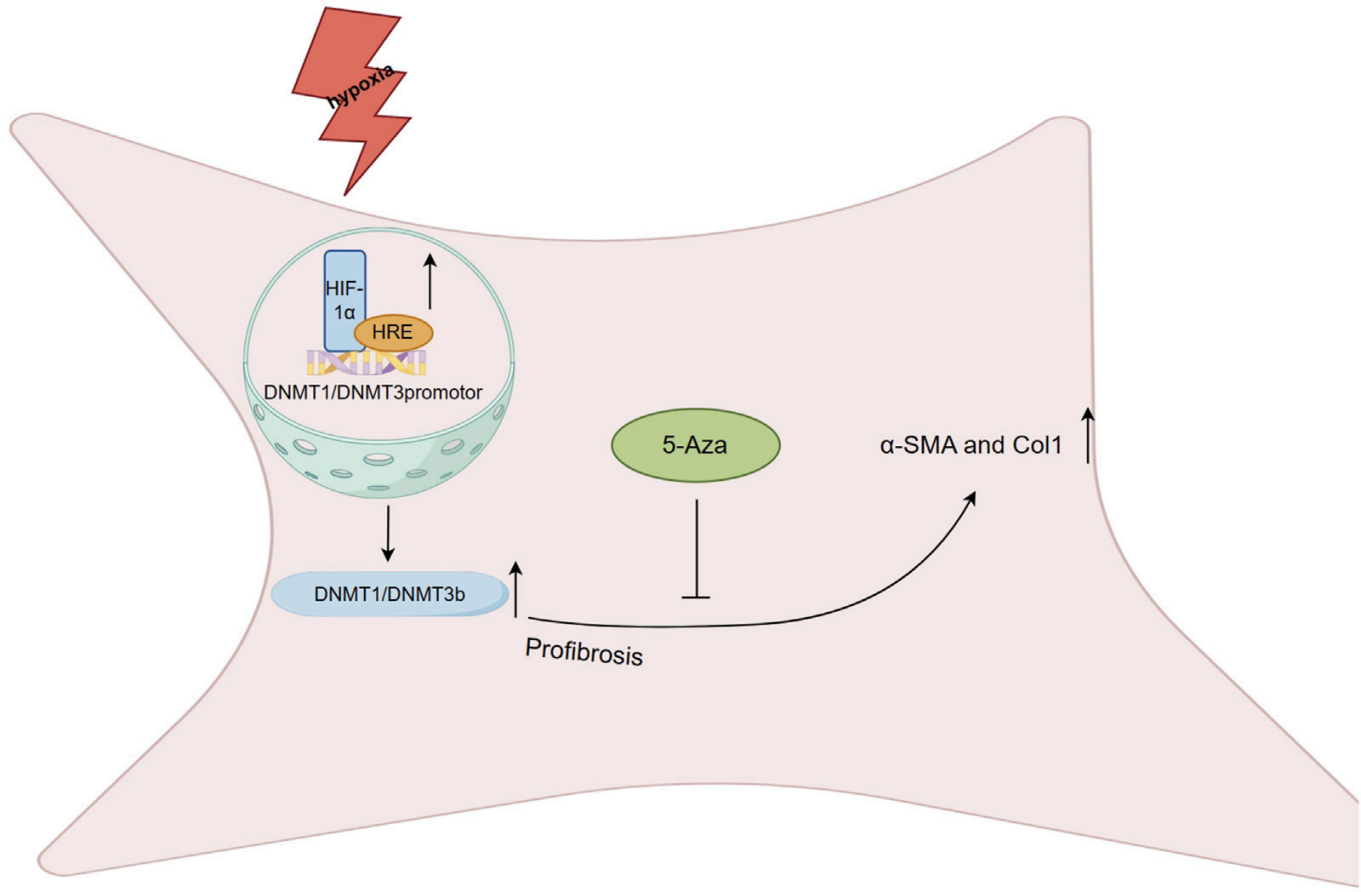
Regulation of DNA methylation on fibrosis after myocardial infarction HIF-1a plays a role in regulating global DNA hypermethylation and a profibrotic state, potentially through the binding of hypoxia response elements (HRE) to DNMT1 and DNMT3b promoter sites, resulting in a significant increase in DNMT1 and DNMT3b expression levels. Under hypoxic conditions, the profibrotic effect of TGF-β can be inhibited by the 5-Aza, which significantly reduces the expression levels of α-SMA and Col1.

After MI, ischemic hypoxic injuries commonly occur and may require long-term adaptation involving epigenetic modifications to maintain the fibrotic phenotype. A study quantified the expression levels of the CAIX gene, regulated by hypoxia-inducible factor-1 (HIF-1), as a means to assess the degree of myocardial tissue hypoxia. Prolonged exposure of human cardiac fibroblasts to 1% hypoxia resulted in a profibrotic state, and the degree of hypoxia correlated with collagen 1 (Col1) and α-SMA expression levels and collagen deposition (Holotnakova et al., 2008). HIF-1a plays a role in regulating global DNA hypermethylation and a profibrotic state, potentially through the binding of hypoxia response elements (HRE) to DNMT1 and DNMT3b promoter sites, resulting in a significant increase in DNMT1 and DNMT3b expression levels (Watson et al., 2014). Moreover, under hypoxic conditions, the profibrotic effect of TGF-β, a profibrotic agonist, can be inhibited by the DNMTi 5-aza-2′- deoxycytidine (5-aza), which significantly reduces the expression levels of α-SMA and Col1 (Figure 4). However, previous studies have shown that TGF-β1 may inhibit DNMT expression levels and overall activity, while upregulating Col1A1 in cardiac fibroblasts. The role of DNA methylation in the fibrotic process *in vivo* during hypoxia induction requires further investigation, but the strong correlation of DNMT1 and DNMT3b in hypoxic injury and fibrosis suggests the potential for investigating basic and fibrosis-targeted therapies (Watson et al., 2014).

The differentiation of cardiac fibroblasts into α-SMA expressing myofibroblasts is a crucial aspect of the fibrotic process after MI. Recent studies have suggested that post-MI α-SMA expression is regulated by DNA methylation, highlighting the importance of both in cardiac fibroblast differentiation (Hinz et al., 2007; Souders et al., 2009; Williams et al., 2014; He et al., 2019). In the infarcted area of MI rats, α-SMA is overexpressed, but DNMT1 expression levels are decreased. *In vitro* treatment of cardiac fibroblasts with TGF-β1 has been shown to induce upregulation of α-SMA expression by significantly inhibiting DNMT1-mediated DNA methylation at multiple CpG sites in the α-SMA promoter. The Smad and MAPK pathways also play a role in regulating DNMT1 expression in cardiac fibroblasts (He et al., 2019).

Although there are still controversies regarding cardiac fibroblast differentiation, it is undeniable that DNA methylation plays an essential role in maintaining the fibrotic phenotype during the profibrotic process after MI. Further research is needed to resolve the existing contradictions and to investigate the specific manifestations of post-MI epigenetic modifications in the fibrotic process (Pan et al., 2013; Watson et al., 2014; He et al., 2019). DNMT-related drug targets hold great potential for regulating expression in both fibrosis and the control of cardiac fibroblast differentiation, making them highly valuable as clinical therapeutic properties (Tao et al., 2014; Watson et al., 2014).

### 2.4 Cardiomyocyte proliferation and DNA methylation

The imbalance of cardiomyocyte death and regeneration is one of the factors contributing to adverse myocardial remodeling and cardiac dysfunction (Gill et al., 2002; van Empel et al., 2005). The heart, being one of the least regenerative organs in the mammalian body, has limited regenerative capacity in the adult period, as cardiomyocytes essentially exit the mitotic cell cycle and stop cell proliferation (Drenckhahn et al., 2008; Walsh et al., 2010; Laflamme and Murry, 2011). This lack of regenerative capability is the basis for functional deficits in the regeneration and repair of the adult heart in response to pathological insults, such as MI, ultimately leading to cardiac dysfunction and HF (Ponnusamy et al., 2019). However, non-mammals such as amphibians and teleosts maintain significant cardiac regenerative capacity throughout life (Oberpriller and Oberpriller, 1974; Jopling et al., 2010; Kikuchi et al., 2010), and adult zebrafish can induce cardiomyocyte proliferation and overcome scar formation after removing 20% of their ventricles, achieving complete cardiac regeneration (Poss et al., 2002). Recent studies have shown that humans and other mammals still maintain some renewal capacity throughout their lifespan, including after MI (Beltrami et al., 2001; Bergmann et al., 2009). A small number of cardiomyocytes undergoing mitotic proliferation have been observed in the infarct border zone after MI in patients (Beltrami et al., 2001). However, eventually, the myocardium does not regenerate appreciably but instead replaces it with a fibrotic scar (Nag et al., 1983). In ischemic heart disease, 152 cardiomyocytes per million undergo proliferation, a tenfold elevation compared with the normal left ventricle (Kajstura et al., 1998). Achieving therapeutic cardiac regeneration, such as activating cardiomyocyte proliferation *in situ*, is currently an important biomedical goal of therapeutic strategies after MI (Karra and Poss, 2017). Nonetheless, strong and precise targeting of cardiomyocyte proliferation is necessary to prevent unintended proliferative events in non-cardiomyocytes and to avoid any neoplastic effects, ensuring safety (Lin and Pu, 2014; Gabisonia and Recchia, 2018).

Cardiac progenitor cells (CPCs) are crucial resident cells of the heart with multipotent, clonogenic, and self-renewal abilities for cardiac regeneration (Urbanek et al., 2006; Sanada et al., 2014; Leri et al., 2015). These cells can differentiate into cardiomyocytes, endothelial cells, and vascular smooth muscle cells, playing a significant role in protecting the heart and generating blood vessels to prevent adverse cardiac remodeling after MI (Beltrami et al., 2003; Matsuura et al., 2009; van Berlo et al., 2014; Liu et al., 2015). However, after MI, the population of distinct cardiac side-population cells is reduced to less than half of their original levels 1 day after the injury, and only a few CPCs remain once large numbers are transplanted to the injured heart (Mouquet et al., 2005; Su et al., 2018). Moreover, patients who suffered from MI showed higher levels of serum HMGB1, a protein closely related to inflammatory response, cell proliferation, and apoptosis (Bell et al., 2006; Goldstein et al., 2006; Gwak et al., 2012; Kang et al., 2014). Studies have reported that CPCs treated with hypoxia significantly increased the level of expressed HMGB1, and knockdown of HMGB1 could attenuate hypoxia-induced apoptosis in these cells. This mechanism was found to be due to the hypoxic stimulus inhibiting the expression of DNMT1. This reduction in DNMT1 led to a decrease in the methylation of CpGi at HMGB1 promoter in CPCs, subsequently upregulating the HMGB1 expression level. Additionally, the MAPK signaling pathway is also involved in regulating the mechanism of post-hypoxic apoptosis of CPCs mediated by HMGB1. This study identified an important role of the MAPKs/DNMT1/HMGB1 signaling axis in regulating post-hypoxic apoptosis of CPCs and provided a new direction for CPCs apoptosis and proliferation, contributing to the development of valuable targets for stem cell therapy after MI (Su et al., 2018).

Numerous studies have demonstrated the involvement of the Notch signaling pathway in processes relevant to the regulation of cardiomyocyte proliferation during heart development (Pedrazzini, 2007; Niessen and Karsan, 2008; de la Pompa and Epstein, 2012; MacGrogan et al., 2018; Wang et al., 2021). In particular, Notch1 is highly expressed in immature proliferating cardiomyocytes during the early neonatal period to protect them from apoptosis (Collesi et al., 2008). Reactivation of the Notch signaling pathway has been shown to induce embryonic stem cell-derived ventricular myocytes to re-enter the cell cycle (Campa et al., 2008). Studies have demonstrated that DNA methylation serves as an irreversible marker of transcriptional repression (Cedar and Bergman, 2009), and after birth in neonatal rats, cardiomyocyte proliferation is reduced coincident with a significant reduction in Notch1 signaling levels (Felician et al., 2014). Yet, utilizing adeno-associated virus vectors for gene transfer to activate the Notch1 signaling pathway after MI in adult mice proves ineffective. Additionally, deliberately activating the Notch1 pathway does not succeed in inducing cardiomyocyte proliferation in adult mice after MI. It has been proposed that the terminal differentiation state of cardiomyocytes is associated with the temporally progressive DNA methylation of Notch1 promoters and its target genes (Hes1, Hey1, and Hey2). Treatment with DNMTi 5-aza has been confirmed to correlate with the repressive role of Notch gene promoter methylation. This study argues that induction of adult cardiomyocyte proliferation by Notch pathway stimulation is not a suitable strategy to promote cardiac regenerative responses in adult mice. However, as research progresses, DNA demethylation-related epigenetic modifications and epigenetic drugs such as DNMTi advance clinical experiments in the field of cancer (Jones et al., 2016; Wu and Zhang, 2017; Soler-Botija et al., 2020). The methylation sites of Notch and its target genes may become highly promising therapeutic targets soon.

**FIGURE 5:**
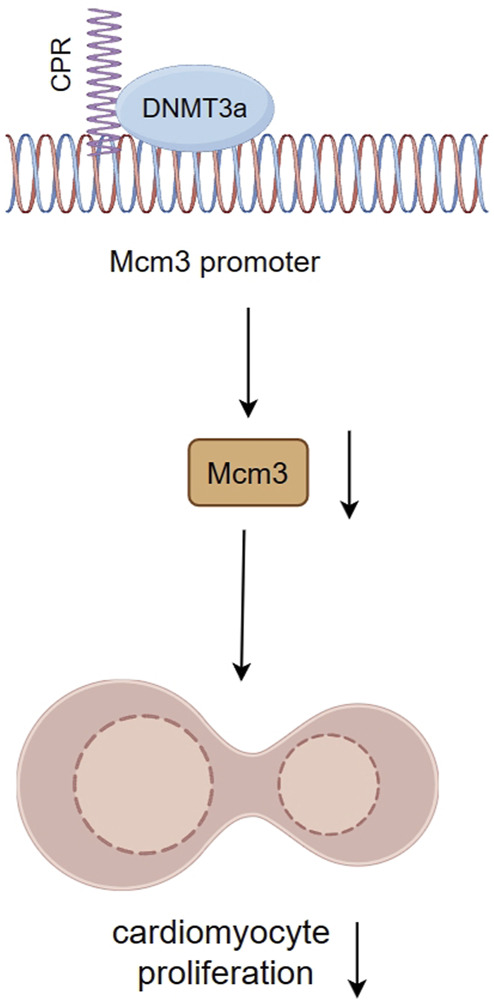
The interaction between CPR and DNMT3a inhibits cardiomyocyte proliferation by inhibiting Mcm3. The interaction of CPR with DNMT3a guides DNMT3a to bind CpGi sites of Mcm3 promoter region, directionally elevating methylation levels, thereby reducing Mcm3 expression and inhibiting cardiomyocyte proliferation.

A long non-coding RNA named cardiomyocyte proliferation regulator (CPR) has potential regulatory effects on cardiomyocyte proliferation and cardiac repair. Studies have found that CPR negatively regulates cardiomyocyte proliferation and regeneration, whereas silencing CPR significantly increases cardiomyocyte proliferation in postnatal and adult hearts and restores cardiac function after MI (Ponnusamy et al., 2019). The interaction of CPR with DNMT3a guides DNMT3a to bind CpGi sites of the minichromosome maintenance protein 3 (Mcm3) promoter region, an initiator of eukaryotic genome replication and cell cycle progression. This targeted binding leads to an increase in methylation levels, subsequently diminishing the expression of Mcm3 and inhibiting cardiomyocyte proliferation (Figure 5). Loss of cardiomyocyte cell cycle activity induces robust fibrosis response and scar formation in the adult heart after injury (Porrello et al., 2011). Aberrant fibrosis, scarring, and loss of cardiac regenerative capacity after MI are all critical contributors to adverse cardiac remodeling. The reduction of methylation levels of the Mcm3 promoter region by targeted inhibition of CPR expression or using epigenetic modifying drugs such as DNMTi may be a constructive view to promote cardiomyocyte proliferation after MI and alleviate adverse cardiac remodeling (Ponnusamy et al., 2019).

Epigenetic reprogramming exhibits great potential after MI, such as transplantation to treat MI by *in vitro* DNA methylation modification, converting bone marrow progenitor cells into cardiac progenitor cells (Rajasingh et al., 2011), and reprogramming of fibroblasts into functional cardiomyocyte-like cells or inducing cardiomyocyte proliferation (Ieda et al., 2010; Liu and Schwartz, 2012; Qian et al., 2012). Through continued research progress, we can bridge the gap in clinical trials related to epigenetic drugs for cardiomyocyte proliferation in cardiovascular diseases and identify the most effective targets to promote cardiac tissue regeneration after MI.

### 2.5 Drug treatment and DNA methylation

Currently, medications such as aspirin, warfarin, tissue plasminogen activator, and interventional therapy, such as percutaneous coronary intervention (PCI), are the mainstay of treatment for AMI (Lu et al., 2015; Doenst et al., 2019; Sabatine and Braunwald, 2021). However, unpredictable complications such as bleeding, ischemia/reperfusion injury, and coronary restenosis may occur, highlighting the need for safer and more innovative therapeutic strategies to optimize clinical outcomes (McCarthy et al., 2018; Doenst et al., 2019; Mackman et al., 2020; Zhang et al., 2022).Several studies have observed significant individual methylation differences in patients with MI (Ek et al., 2016; Rask-Andersen et al., 2016; Thunders et al., 2019; Han et al., 2022; Luo et al., 2022; Ren et al., 2022). Further exploration of the regulatory mechanisms of DNA methylation and its potential impact on metabolism and vascular physiology after MI may help reduce the incidence of post-MI complications (Ward-Caviness et al., 2018). Epigenetic modification-related drugs, particularly DNMT inhibitors, have demonstrated efficacy in the treatment of acute myeloid leukemia and myelodysplastic syndrome (Sudan et al., 2006; Cashen et al., 2010; Lübbert et al., 2012; Saunthararajah et al., 2012). Although the research on DNA methylation-related drugs in cardiovascular diseases is relatively lacking, the existing achievements reflect considerable value and promise, given the success of epigenetic drugs in other fields such as cancer.

Clopidogrel is a medication that significantly reduces the risk of adverse ischemic events after PCI compared to aspirin (Steinhubl et al., 2002). However, resistance to clopidogrel remains an important factor contributing to the recurrence of ischemic events following antiplatelet therapy (Mehta et al., 2001). Recent studies have shown that clopidogrel resistance is associated with increased DNA methylation levels at the promoters of genes such as ABCB1, P2Y12, and PON1 (Su et al., 2014; Su et al., 2017; Su et al., 2019). Furthermore, CYP2C19, a key enzyme in the biotransformation of clopidogrel, and its reduction in DNA methylation have been found to increase the risk of resistance and worsen clinical outcomes in STEMI patients undergoing clopidogrel preconditioning after PCI (Sukmawan et al., 2021). All 14 CpGis of the CYP2C19 gene are located in the gene body, which, according to the methylation paradox, suggests that reduced DNA methylation levels in the CYP2C19 gene body are responsible for its reduced transcriptional expression, according to the methylation paradox (Jones, 1999). The reduction in CYP2C19 levels blocks the biotransformation of clopidogrel, reducing active metabolites, which leads to resistance. Genetic polymorphisms of CYP2C19 have also been found to increase the risk of resistance to clopidogrel by 4.2 to 5.3-fold (Sukmawan et al., 2021). Recent advances in CYP2C19 DNA methylation and clopidogrel resistance further point to epigenetic modifications playing a key role in post-MI pharmacotherapy.

Effective mitigation strategies are urgently needed for H/R injury caused by post-MI treatment (Hausenloy and Yellon, 2013). Dexmedetomidine (DEX), a highly selective α2-adrenoceptor agonist commonly used as a sedative and anesthetic agent in clinical settings, has been extensively studied for its protective effects against H/R-mediated organ injuries, including those affecting the brain, liver, and kidney (Yuan et al., 2019; Yu et al., 2020; Zhang B. et al., 2021; Wu et al., 2021). DEX has been shown to inhibit cardiomyocyte apoptosis triggered by H/R injury by upregulating SIRT1 via the SIRT1/CHOP pathway (Zhang Y. et al., 2021). Overexpression of SIRT1 can hinder NF-κB acetylation, thus improving cardiac function (Lin et al., 2020). Moreover, a recent study has refined and expanded on these findings by exploring the link between DNA methylation and H/R injury. By increasing SIRT1 expression levels through DNMTi-mediated demethylation of the SIRT1 promoter, DEX can inhibit NF-κB activation and ameliorate H/R-triggered myocardial injury (Wang et al., 2022). Given that Tet1-mediated DNA demethylation is critical to DEX’s mechanism of action in alleviating H/R injury, this approach represents a promising new pharmacological strategy based on DNA methylation for mitigating H/R injury after MI.

Although progress in epigenetic drug applications for MI-related cardiovascular diseases in clinical trials has been slow, preclinical studies in animal models have shown promising results (Soler-Botija et al., 2020). For instance, in a rat MI model, the DNA methylation inhibitor 5-AZ was found to shift macrophages towards an M2 anti-inflammatory phenotype and prevent cardiac contractile decompensation by inhibiting the promoter activity of inducible nitric oxide synthase (iNOS). Furthermore, 5-AZ was found to activate ubiquitin-conjugating enzyme 9 to mediate the sumoylation of interferon regulatory factor 1 in macrophages, which circumvented rapid degradation by ubiquitination in the proteasome and enabled its accumulation in cells (Nakagawa and Yokosawa, 2000). By altering its effect and incapacitating iNOS, 5-AZ promotes macrophages to switch to an M2 anti-inflammatory phenotype (Jeong et al., 2015). Given the clinical availability of 5-AZ as an epigenetic drug, it may offer a promising new direction for post-MI inflammation therapy. In an MI rat model with reperfusion injury, treatment with another DNMTi, epigallocatechin-3-gallate (EGCG), reduced plasma interleukin-6 levels, and post-ischemic neutrophil infiltration. EGCG suppressed IκB kinase (IKK) activity after reperfusion and markedly weakened the degradation of inhibitor κB-α, thereby significantly reducing NF-κB’s bound state to DNA. Moreover, EGCG attenuated c-Jun phosphorylation at all time points post-MI reperfusion, affecting the activation process of AP-1 (Aneja et al., 2004). These findings suggest that EGCG may inhibit the IKK/NF-κB signal transduction pathway directly or indirectly by altering the redox status at the site of inflammation. By leveraging its properties as an epigenetic drug and understanding its underlying molecular mechanism, EGCG may offer a promising clinical therapeutic strategy for modulating the associated inflammation during blood flow reconstruction after MI.

We summarize the DNA methylation drugs mentioned above and the DNA methylation targets involved in the pathophysiological processes after myocardial infarction in Table 1; Supplementary Table S1, respectively.

**TABLE 1:** DNA methylation drugs in related research after myocardial infarction.

Type	Target	Medication	Model	Epigenetic mechanism	References
DNA methyltransferase inhibitors (DNMTi)	DNMTs	5-Aza	Myocardial infarction in rats	Inhibited iNOS promoter activity and shifted macrophages to the M2 phenotype following MI attenuating the pro-fibrotic behaviors of stimulated cardiac fibroblasts	Kim et al. (2014)
			Myocardial infarction in rats	Potentiated IRF1 sumoylation repressing transcriptional activity for iNOS and allowed macrophages to shift to the M2 phenotype resulting in resolving post-MI inflammation	Jeong et al. (2015)
			human cardiac fibroblast cells	Application of the DNMTi suppressed the pro-fibrotic effects of TGFβ	Watson et al. (2014)
		Epigallocatechin-3-gallate (EGCG)	Myocardial Ischemia reperfusion injury in rats	Reduced myocardial damage, myeloperoxidase activity, plasma IL-6 and creatine phosphokinase levels associated with a specific inhibition of the IKK/NF-κB and AP-1 pathway to protect against myocardial reperfusion injury	Aneja et al. (2004)
Tet methylcytosine dioxygenase 1 (TET1) activator	TET1	Dexmedetomidine (DEX)	Myocardial Ischemia reperfusion injury in rats	Upregulated Sirt1 promoter expression by fostering TET1-mediated demethylation thereby restrained the activation of NF-κB to improve H/R-mediated myocardial injury	Wang et al. (2022)

## 3 Conclusion

Considerable *in vivo* and *in vitro* evidence now supports the potential use of epigenetic drugs as therapeutic tools to improve functional recovery after MI, however, there is currently no corresponding clinical evidence. Conducting clinical trials with a limited number of patients can lay the groundwork for identifying safe and efficacious epigenetic drugs, paving the way for their broader application in a larger patient population. Currently, epigenetic drugs in clinical trials are broadly divided into two classes: genomic drugs, such as the DNMTi mentioned, for extensive reprogramming, and targeted precision medicine for the treatment of specific diseases. DNMTi, in particular, have shown great promise in post-MI treatment, suggesting their potential for further development in clinical applications. We believe that drugs targeting the prevention of cardiomyocyte death and promotion of cardiomyocyte regeneration during the process of myocardial infarction may have promising clinical prospects. However, many drugs, such as DNMTi, are administered systemically, and their side effects require further evaluation. With the development of targeted drug delivery technologies, these drugs may be widely applied in the future.
