# Supplementary material for: A review on regulation of DNA methylation during post-myocardial infarction
Source: Front Pharmacol. 2024 Feb 13;15:1267585. doi: 10.3389/fphar.2024.1267585 (PMC10896928; doi:10.3389/fphar.2024.1267585)
Supplement: Supplementary file 1 [file Table2.DOCX]

| **Table S1: Targets related to DNA methylation in the repair process after myocardial infarction** | | | | |
| --- | --- | --- | --- | --- |
|  | **Target** | **Associated Function** | **Epigenetic Mechanism** | **References** |
| **Inflammation** | Inducible nitric oxide synthase (iNOS) promoter | Implicated in the classic activation of macrophages. | Improved iNOS promoter activity by promoting methylation to shift macrophages to the M1 phenotype following MI and enhanced the pro-fibrotic behaviours of stimulated cardiac fibroblasts. | **68** |
|  | Runt-related transcription factor (RUNX)3 promoter | Involved in the immune system and inflammatory pathways and regulated the hypoxia-induced endothelial-to-mesenchymal transition of human cardiac microvascular endothelial cells (CMECs). | Down-regulated RUNX3 expression through promoting RUNX3 methylation caused by lncRNA KCNQ1OT1 recruiting DNMT1 and offered protection against CMEC injury and inflammatory response in AMI. | **77** |
|  | Spleen focus forming virus proviral integration oncogene (SPI1) promoter | Identified as one of the key factors involved in the development of post-MI heart failure which was correlated with inflammation, immune activity, and cell apoptosis. | Upregulated SPI1 by reducing DNA methylation and augmented development of myocardial infarction through activating the TLR4/NFκB axis. | **81** |
| **Autophagy** | Phosphatase and tensin homolog-induced putative kinase 1 (Pink1) promoter | Downregulated during human end-stage heart failure suggesting that Pink1 was essential for normal heart function. | Activated Pink1 expression through autophagy-related circular RNA (ACR) directly binding to Dnmt3B and phosphorylated FAM65B inhibiting autophagy and cell death in the heart. | **101** |
|  | MiR-30a promoter | Abnormally expressed in the cardiac tissue of murine model with myocardial I/R and the regulation of miRNAs on autophagy was mediated by autophagy-related genes such as BECN1, light chain 3B (LC3B) and p62. | Upregulated miR-30a by DNMT3b mediating hypomethylation in the cardioprotective effect of HPostC thereby suppressing BECN1 expression and attenuating autophagy to survival against hypoxia/reoxygenation (H/R) injury. | **114** |
|  | DNA damage regulated autophagy modulator 1 (DRAM1) promoter | Increased expression locally in the heart infarct border was protective against adverse LV remodeling and restored autophagy flux after AMI. | Downregulated DRAM1 by promoting CpG sites methylated and through DRAM1-Atg7-Atg12/Atg5 autophagy flux regulation pathway to protect cardiomyocytes from ischemia stress-induced autophagy flux obstacle. | **118** |
| **Fibrosis** | Nei like DNA glycosylase 3 (NEIL3) | Played a role in the regulation of cell proliferation and genetic variant of human NEIL3 was associated with increased risk of MI. | Modulated the 5hmC levels and the balance between epigenetic methylation of cytosine and oxidative demethylation to regulate proliferation and differentiation of fibroblast-like cells during MI repair. | **135** |
|  | Hypoxia inducible factor-1(HIF-1a) | Prolonged local tissue hypoxia lead to aberrant ventricular remodeling and cardiac fibrosis likely attributed to the modification of chromatin structure. | Resulted in aberrant DNA hypermethylation by binding hypoxia response element (HRE) with promoters of DNMT1 and DNMT3b to contribute to the fibrotic burden associated with both collagen 1 and ASMA expression. | **136** |
|  | α‐Smooth muscle actin (α‐SMA) promoter | Differentiated to myofibroblasts expressing α‐SMA was crucial in cardiac fibrosis and DNMT‐mediated DNA methylation of the α‐SMA promoter contributed to lung fibroblast differentiation. | Activated α‐SMA expression in the induction of myofibroblast differentiation via removing methylation established by DNMT1 additionally involved with the Smad and MAPK pathways. | **152** |
| **Cardiomyocyte Proliferation** | High-mobility group box 1 (HMGB1) promoter | Associated with the inflammatory response, cell proliferation and apoptosis, angiogenesis, and cell growth such as involving in Jurkat cell and hepatocyte apoptosis. | Upregulated HMGB1 in CPCs controlled by DNMT1 via changing the methylation level of CpGs under hypoxia conditions additionally involved with MAPK pathway and contributed to the apoptosis and proliferation of CPCs. | **180** |
|  | Notch1 promoter | Acted as a key to sustain proliferation of a pool of still immature cardiomyocytes and exerted beneficial effect after heart injury. | Progressive DNA methylation status of Notch1 and its target genes correlated with terminal differentiation of cardiomyocytes. | **193** |
|  | Minichromosome maintenance protein 3 (MCM3) promoter | As a prominent target of lncRNA cardiomyocyte proliferation regulator (CPR) whose absence remarkably improved cardiac function and tissue repair after MI injury. | Silenced MCM3 expression caused by lncRNA CPR interacting and recruiting DNMT3A and led to suppress cardiomyocyte proliferation. | **160** |
